# Prevalence of abdominal aortic aneurysms – a screening program in São Paulo, Brazil

**DOI:** 10.1590/S1516-31802004000400005

**Published:** 2004-07-01

**Authors:** Pedro Puech-Leão, Lazlo Josef Molnar, Ilka Regina de Oliveira, Giovanni Guido Cerri

**Keywords:** Aortic aneurysm, Aneurysm, Screening, Prevalence, Determination, Aneurisma aórtico, Detecção, Prevalência, Aneurisma, Peneiramento

## Abstract

**CONTEXT:**

Abdominal aortic aneurysm is an asymptomatic but potentially fatal condition. Elective surgery can prevent death from rupture, and is indicated for aneurysms larger than 45 mm. Because aneurysms tend to grow with time, detection of small ones (> 29 mm) may lead to a closer follow-up of patients at risk.

**OBJECTIVE:**

To determine the prevalence of abdominal aortic aneurysms in São Paulo, Brazil.

**DESIGN:**

Prospective, descriptive.

**SETTING:**

University Hospital.

**PARTICIPANTS:**

Persons aged 50 years or more were offered, through the press, the opportunity to be screened for abdominal aortic aneurysm. The total number screened was 2,756.

**PROCEDURE:**

All were submitted to abdominal palpation and ultrasound examination.

**PARAMETER STUDIED:**

A maximum diameter of 30 mm or more was considered to be an aneurysm.

**RESULTS:**

Sixty-four aneurysms were detected, nine of which measuring more than 49 mm. Palpation detected 60 aneurysms, but only 20 of these were confirmed by the ultrasound. Conversely, 41 of the ultrasound-detected aneurysms were not palpable. The percentages of abdominal aortic aneurysms found in the subgroups via ultrasound examination (with 95% confidence interval) were as follows: total group, 2.3 (1.8-3); men, 4.6 (3.5-5.9); women, 0.6 (0.3-1.1); men aged 60 or more, 6 (4.3-8); women aged 60 or more, 0.9 (0.4-1.8).

**CONCLUSION:**

In São Paulo, Brazil, 1.8 to 3 % of persons aged 50 years or more are expected to have abdominal aortic aneurysms. In the subgroup of men aged 60 or more, the expected prevalence is between 4.3 and 8%.

## RESUMO

**CONTEXTO:**

O aneurisma da aorta abdominal é uma doença assintomática, mas potencialmente fatal. Correção cirúrgica eletiva pode prevenir a rotura, e está indicada para aneurismas maiores do que 49 mm. Uma vez que os aneurismas tendem a crescer ao longo do tempo, a detecção daqueles pequenos implica em um acompanhamento cuidadoso destes pacientes.

**OBJETIVO:**

Conhecer a prevalência do aneurisma da aorta abdominal na população de São Paulo, Brasil.

**DESENHO:**

Estudo prospectivo, descritivo.

**LOCAL:**

Hospital Universitário da Universidade de São Paulo.

**PARTICIPANTES:**

Foi oferecida, por meio da imprensa, a indivíduos com 50 anos ou mais, a oportunidade de serem examinados para detecção de aneurisma da aorta abdominal. O número total de pessoas examinadas foi 2.756.

**PROCEDIMENTO:**

Todos foram submetidos a palpação abdominal e a ultra-sonografia.

**VARIÁVEIS ESTUDADAS:**

Um diâmetro máximo de 30 mm ou mais foi considerado aneurisma.

**RESULTADOS:**

64 aneurismas foram detectados, 9 dos quais medindo mais de 49 mm. A palpação detectou 60 aneurismas, mas apenas 20 destes foram confirmados pela ultra-sonografia; por outro lado, 41 dos aneurismas detectados pela ultra-sonografia não eram palpáveis. A percentagem de aneurismas da aorta abdominal encontrados na ultra-sonografia nos subgrupos e o intervalo de confiança de 95% foram os seguintes: total, 2,3 (1,8-3); homens, 4,6 (3,5-5,9); mulheres, 0,6 (0,3-1,1); homens com 60 anos ou mais, 6 (4,3-8); mulheres com 60 anos ou mais, 0,9 (0,4-1,8).

**CONCLUSÃO:**

Em São Paulo, Brasil, estima-se que 1,8 a 3% das pessoas com 50 anos ou mais tenham aneurisma da aorta abdominal. No subgrupo de homens com 60 anos ou mais, a prevalência está entre 4,3 e 8%.

## INTRODUCTION

Abdominal aortic aneurysm is an asymptomatic but potentially fatal condition. Diagnosis is usually made during image testing for other diseases. Because death can be prevented by elective surgical intervention, screening is of utmost importance, just as it is for prostate or breast cancer.

For the establishment of public health policies, it is important to know the prevalence of aortic aneurysms in a given population, and especially in subgroups of the population, in order to focus attention on that particular group. Given the fact that this disease has familial incidence, its prevalence may vary between countries and from one ethnic group to another.

The aim of this study was to determine the prevalence of abdominal aortic aneurysms in a non-hospital population in São Paulo, Brazil.

## METHOD

During the month of September 1999, an announcement that Hospital das Clínicas was offering free ultrasound scans for abdominal aortic aneurysms was broadcast on radio and TV stations in São Paulo. Anybody aged 50 years or more was invited to call a special telephone number and make an appointment. Brief appearances by members of the Vascular Surgery Division were sometimes included in the broadcasts, explaining what the disease is about, and the need for screening. Additionally, outdoor banners were placed in the neighborhood of the hospital, and an announcement was added to every water bill in the city. No announcement was made to the public inside the hospital, because the aim was to screen the healthy population and not those being treated for any other disease.

The first 3,000 persons to call were placed on the schedule. Upon arrival at the hospital, the subject was asked if he (or she) had been ever examined for aortic disease, and received the information in writing that he (or she) would be submitted to ultrasonography of the abdominal aorta. The text emphasized the fact that no other abdominal organs would be studied, and also that thoracic aortic aneurysms would not be detected.

Palpation of the abdomen was performed by vascular surgeons, with the patient in the supine position. The aortic pulse was sought and the size of the aorta was described as normal or dilated. Ultrasonography of the abdominal aorta was conducted by radiologists, in two rooms equipped with ultrasound systems (Logic 400, General Electric 3.5 MHz curved array probes). The probe was positioned over the projection of the aorta, and its full extent from the infrarenal segment to the bifurcation was examined. No other organs or structures were examined. The maximum infrarenal diameter was measured in all cases, except for those whose aortic imaging was not clear enough. With the help of dedicated nursing staff, the average number of examinations per hour was 32.

For the purposes of this study, an aneurysm was defined as a dilatation of more than 29 mm in diameter. Aneurysms with diameters between 30 and 49 mm were classified as small, and those exceeding 49 mm as large. Patients found to have small aneurysms were advised to have the test repeated in six months, and those with aortic diameters of more than 49 mm were registered for pre-operative evaluation and surgical correction.

## RESULTS

Palpation and ultrasound were performed on 2,799 subjects. Some of those scheduled did not show up, and some came without scheduling and were examined as well. Thirty persons aged less than 50 years came, despite the specificity of the announcements: they were examined as a courtesy, but not included in the final count (and none of these persons had aneurysms). One patient declared that he had a small aneurysm that was under surveillance by his physician and was taking the opportunity for a free check on its diameter: he was also not included in the count, although examined. In twelve cases the aortic image was not adequate for measurement, due to either obesity or excess of air in the intestines. Hence, 2,756 cases were considered as the population sample for statistics.

Sixty-four aneurysms were found in this survey. The distribution according to age and gender is shown in Tables 1 and 2. In the female group, nine aneurysms were found, seven in women aged more than 60 years. The only two large aneurysms found in women were also in this age group.

**Table 1:** Number of aneurysms (aorta > 30 mm from ultrasound) according to age and gender in a screening study carried out in São Paulo.

Population	Total (%)	Aneurysms found (%)	95% CI
All	2,756 (100)	64 (2.32)	1.79 – 2.96
age > 60	1,517 (55)	50 (3.30)	2.45 – 4.33
age > 65	947 (34.3)	37 (3.91)	2.77 – 5.36
Male	1,228 (44.5)	56 (4.56)	3.46 – 5.88
male, age > 60	722 (26.1)	43 (5.96)	4.34 – 7.95
Female	1,528 (55.44)	9 (0.59)	0.27 – 1.11
female, age > 60	795 (28.8)	7 (0.88)	0.35 – 1.80

*CI = confidence interval.*

**Table 2:** Number of large aneurysms according to age and gender (aorta ≥ 50 mm at ultrasound) in a screening study carried out in São Paulo

Population	Total (%)	Aneurysms found (%)	95% CI (%)
All	2,756 (100)	9 (0.33)	0.15 – 0.62
age > 60	1,517 (55)	8 (0.53)	0.23 – 1.04
age > 65	947 (34.3)	7 (0.74)	0.30 – 1.50
Male	1,228 (44.5)	7 (0.57)	0.23 – 1.17
male, age > 60	722 (26.1)	6 (0.83)	0.31 – 1.80
Female	1,528 (55.44)	2 (0.13)	0.02 – 0.40
female, age > 60	795 (28.8)	2 (0.25)	0.03 – 0.91

*CI = confidence interval.*

Palpation detected 60 aneurysms, but only 20 of these were confirmed by the ultrasound. Conversely, 41 of the ultrasound-detected aneurysms were not palpable (Table 3). Ultrasound examination was shown to be more accurate than palpation in the detection of the disease.

**Table 3:** Results of palpation and corresponding confirmation of aneurysms of abdominal aorta by ultrasound examination in a screening study carried out in São Paulo (n = 2,756)

Palpation	Number of patients	Aneurysm confirmed by ultrasound
Impossible	298	3
Negative	2,398	41
Positive	60	20

## DISCUSSION

The prevalence of abdominal aortic aneurysms in the population of southeastern Brazil, as estimated from this sample population, is similar or slightly larger than that of other series (Table 4),^1-13^ except for that of Bonamigo and Siqueira,^13^ the only other survey in the Brazilian population. We could not find a reasonable explanation for this discrepancy.

**Table 4:** Results of screening for abdominal aortic aneurysm in the literature and our data

Author	Country	Population Gender/age	Number screened	Criteria for positiveness (diameter in mm)	% of patients with aneurysm
Holdsworth et al.^1^	United Kingdom	Men/65-79	628	> 29	6.7
Kyriakides et al.^2^	United Kingdom	Men/65	3,497	> 30	4.9
Vazquez et al.^3^	Belgium	Men/65 and 75	727	> 29	4.5
Boll et al.^4^	Netherlands	Men/65-80	2,416	> 29	8.1
Scott et al.^5^	United Kingdom	Men/65-80	1,947	> 29	7.6
		Women/65-80	2,290	> 29	1.3
Lindholt et al.^6^	Denmark	Men/65-73	3,748	> 29	4.1
Krohn et al.^7^	Norway	Men/> 60	500	> 29 or 50% greater than proximal aorta	8.2
Collin et al.^8^	United Kingdom	Men/65-74	426	> 40 or 5 mm greater than suprarenal aorta	5.4
Lederle et al.^9^	United States of America	Men/50-79	52,745	> 29	3.6
Lucarotti et al.^10^	United Kingdom	Men/65	4,232	26-39	7.1
				> 39	1.3
Ögren et al.^11^	Sweden	Men/74	343	> 35	11.1
Morris et al.^12^	United Kingdom	Men/> 50	3,030	> 25	11.1
Bonamigo and Siqueira^13^	Brazil	Men/> 54	1,012	> 29 or 3.5 mm greater than suprarenal aorta	1.7
Present study	Brazil	Men/> 50	1,228	> 29	4.6
		Women/> 50	1,528	> 29	0.6

The aim of this study was to determine the prevalence of the disease in the Brazilian population, since there was a lack of data to guide public health policies. The need for future nationwide screening programs has to be analyzed on the basis of efficiency, efficacy and cost.

Much has been written against screening for aortic aneurysms. The points emphasized are: most aneurysms do not require surgical intervention; aneurysms prevail in an age group close to average life expectancy, thus many patients will die from other causes; and detection of a small aneurysm does not indicate a life threatening situation, but such detection may cause psychological stress.

The need for population screening, however, is reinforced by the fact that aortic aneurysms are asymptomatic, potentially lethal and frequently missed at routine medical examinations. Karkos et al., in analyzing the charts from 198 patients who presented with this diagnosis, reported that only 48% had been discovered clinically, while 37% were found at radiological examinations for other diseases and 14% during laparotomy for other procedures.^14^

The decision to screen the whole population of a given country for abdominal aortic aneurysms should be made on the basis of cost-effectiveness. When the cost is covered by governments, priorities have to be decided on the basis of the total budget and the need for screening of other diseases. On an individual basis, however, we must state that each person has the right to know what kind of disease may possibly affect him, and to decide whether to be screened or not, at his own expense.

## CONCLUSION

The prevalence of abdominal aortic aneurysm in the population of São Paulo aged over 50 years is between 1.9 and 2.96%. In males who are older than 60 years it is estimated to be 4.34 to 7.95%.
